# Impact of Nutrition on Pulmonary Arterial Hypertension

**DOI:** 10.3390/nu12010169

**Published:** 2020-01-07

**Authors:** María Callejo, Joan Albert Barberá, Juan Duarte, Francisco Perez-Vizcaino

**Affiliations:** 1 Department of Pharmacology and Toxicology, School of Medicine, Universidad Complutense de Madrid, 28040 Madrid, Spain; maria.callejo@ucm.es; 2 CIBER Enfermedades Respiratorias, Ciberes, 28029 Madrid, Spain; jbarbera@clinic.cat; 3 Instituto de Investigación Sanitaria Gregorio Marañón (IISGM), 28007 Madrid, Spain; 4 Department of Pulmonary Medicine, Hospital Clínic-Institut d’Investigacions Biomèdiques August Pi i Sunyer (IDIBAPS), Universitat de Barcelona, 08036 Barcelona, Spain; 5 Department of Pharmacology, School of Pharmacy, Universidad de Granada, 18071 Granada, Spain; jmduarte@ugr.es; 6 CIBER Enfermedades Cardiovasculares, CiberCV, 28029 Madrid, Spain; 7 Instituto de Investigación Biosanitaria (ibs.Granada), 18012 Granada, Spain; 8 Centro de Investigaciones Biomédicas (CIBM), 18016 Granada, Spain

**Keywords:** pulmonary hypertension, microbiota, vitamin C, vitamin D, iron, diet

## Abstract

Pulmonary arterial hypertension (PAH) is characterized by sustained vasoconstriction, vascular remodeling, inflammation, and in situ thrombosis. Although there have been important advances in the knowledge of the pathophysiology of PAH, it remains a debilitating, limiting, and rapidly progressive disease. Vitamin D and iron deficiency are worldwide health problems of pandemic proportions. Notably, these nutritional alterations are largely more prevalent in PAH patients than in the general population and there are several pieces of evidence suggesting that they may trigger or aggravate disease progression. There are also several case reports associating scurvy, due to severe vitamin C deficiency, with PAH. Flavonoids such as quercetin, isoflavonoids such as genistein, and other dietary polyphenols including resveratrol slow the progression of the disease in animal models of PAH. Finally, the role of the gut microbiota and its interplay with the diet, host immune system, and energy metabolism is emerging in multiple cardiovascular diseases. The alteration of the gut microbiota has also been reported in animal models of PAH. It is thus possible that in the near future interventions targeting the nutritional status and the gut dysbiosis will improve the outcome of these patients.

## 1. Pulmonary Hypertension

The pulmonary circulation in healthy individuals is a high flow, low resistance circuit. It accommodates a similar cardiac output as the systemic circulation but with one sixth of its pressure. Normal mean pulmonary arterial pressure (mPAP) at rest is 14.0 ± 3.3 mmHg, with an upper limit of normal of 20 mmHg [1]. Pulmonary hypertension (PH) is due to a rise in pulmonary vascular resistance and mPAP. It is a chronic vascular disorder resulting in progressive right heart failure and eventually death [2,3]. A clinical classification categorizes PH into five groups according to their pathophysiological mechanisms, clinical presentation, hemodynamic characteristics, and treatment strategy [1,3]. Group 1, Pulmonary Arterial Hypertension (PAH) is also subclassified into idiopathic, familial, associated with other disorders or infections, or resulting from drug or toxin exposure [3,4]. The definition of PAH has been revised in the 6th World Symposium on Pulmonary Hypertension. PAH is now defined as a mPAP > 20 mmHg at right heart catheterization, normal left atrial pressure, and pulmonary vascular resistance ≥ 3 Wood units [1]. In Europe, PAH prevalence is in the range of 15–60 subjects per million population and an incidence of 5–10 cases per million per year [5,6]. In addition to poor prognosis, with one- and three-year survival rates around 87% and 67%, respectively, limitations in functional status affect the patient’s quality of life, daily life activities, and employment [7,8].

### 1.1. Etiology

Several genetic and environmental factors for the development and progression of PAH have been identified [3,4,9]. In the West, idiopathic PAH, i.e., without any familial history or known triggering factor, is the most common subtype (30–50% of all cases of PAH), followed by connective tissue disease-associated PAH, congenital heart disease-associated PAH, and heritable PAH [5]. Mutations in *BMPR2* (bone morphogenetic protein receptors type II) can be detected in approximately 70% of cases of heritable PAH and they are also identified in 10–20% of IPAH [10]. In addition, mutations in other genes related to BMPR2 signaling axis have been discovered [9]: *ACVRL1/ALK1* (Activin receptor-like kinase 1), *ENG* (endoglin), and *SMAD9* (decapentaplegic homolog 9) [9,11]. Mutations in the *KCNK3* gene, which encodes the potassium channel TASK-1 [12], and in *KCNA5,* which encodes the voltage-dependent potassium channel Kv1.5, have also been identified in PAH patients [13]. Numerous drugs and substances have been involved in the development of PAH, including anorexigens, selective serotonin reuptake inhibitors, interferons, antiviral therapies, chemotherapeutic agents, and tyrosine kinase inhibitors such as dasatinib [3,14]. Finally, PAH is also associated with other systemic disorders, such as connective tissue diseases and portal hypertension, and infections, such as HIV and schistosomiasis [3]. In summary, with the exception of idiopathic PAH, in all forms of the disease, there is a factor known to be involved in its etiopathogeny, including mutations, systemic diseases, congenital heart defects, infections, drugs, and toxins. However, none of them by itself can trigger the disease and the need for a second hit has been proposed. For instance, *BMPR2* mutations present low penetrance: only 42% of the women and 14% of the men carrying the mutation develop the disease [11,15]. Similarly, about 30% of patients with scleroderma and 0.5% of HIV patients develop it [16,17].

### 1.2. Pathophysiology

The main pathophysiological mechanisms of PAH are sustained vasoconstriction, endothelial dysfunction, pulmonary vascular remodeling, in situ thrombosis, and inflammation [2,18,19]. Sustained vasoconstriction and endothelial dysfunction are due to an altered production of endothelial vasoactive mediators. These include decreased vasodilator and antiplatelet factors such as nitric oxide (NO) and prostacyclin (PGI_2_), and increased vasoconstrictors and/or prothrombotic factors such as endothelin-1 (ET-1), serotonin (5-HT), thromboxane (TXA_2_), angiotensin II (Ang II), and diverse growth factors, which also contribute to a hyperproliferative and procoagulant state. Ionic remodeling is also a key feature of PAH. The downregulation of voltage potassium channels, notably Kv1.5 [20,21] and TASK-1 [22,23], results in a more depolarized membrane potential in pulmonary arterial smooth muscle cells (PASMC) in PAH patients, leading to increased intracellular calcium and consequently PASMC vasoconstriction and also PASMC proliferation. Excessive smooth muscle proliferation and resistance to apoptosis due to paracrine growth factors, dysregulation of BMPR2 signaling pathway, dysfunctional potassium channels, and rise of anti-apoptotic proteins, among other factors, lead to smooth muscle hyperplasia. These deranged processes culminate in the obliteration of the pulmonary artery by enlarged intima and media layers [18,24] and the formation of proliferating vascular structures called plexiform lesions [24,25]. Thrombotic events in situ are frequent in PAH and contribute to the narrowing of pulmonary arteries too [19]. Altered immune mechanisms also play a significant role in the pathogenesis of PAH. Pulmonary vascular lesions in PAH patients and animal models reveal a recruitment of inflammatory cells as T- and B-lymphocytes, macrophages, dendritic cells, and mast cells [2,18]. In addition, there is an abnormal circulating level of certain cytokines, such as IL-1β, IL-6, IL-17, TNF-α, and CCL5. Notably, some of these cytokines correlate with a worse prognosis in PAH patients [26].

### 1.3. Current Pharmacological Therapies

Over the last decades, intensive research on the cellular and molecular mechanisms and signaling pathways has provided a better understanding of the pathophysiology of PAH and consequently the identification of different pharmacological treatments. Unfortunately, a definitive cure does not exist for PAH. Currently, the five classes of therapies approved for PAH target the Ca^2+^ entry and the three main dysfunctional endothelial pathways: NO, prostacyclin, and endothelin-1 pathways [27,28]. Inhibitors of cyclic nucleotide phosphodiesterase type 5 (PDE-5), sildenafil and tadalafil, potentiate the action of endogenous NO and promote vasodilation [5,27,28]. Soluble guanylate cyclase (sGC) also acts in the NO signaling pathway catalyzing the transformation of GTP to cGMP. The sGC stimulator riociguat promotes the synthesis of cGMP favoring vasodilation and inhibiting cell proliferation. The action of riociguat is independent of the NO availability. Available prostacyclin-related therapies include synthetic (epoprostenol), prostacyclin analogs (treprostinil and iloprost) and the prostacyclin receptor agonist selexipag [27,28]. Endothelin-1 receptor antagonists (ERAs) include bosentan, macitentan, and ambrisentan [5,27,28].

Despite the current approved drugs as monotherapy have shown a favorable impact on clinical, functional, and hemodynamic outcomes, disease progression is frequently observed. At the 5th World Symposium of PH and based on the high level of evidence gathered from numerous randomized, controlled trials, the use of sequential combination therapy was proposed, at least in PAH patients with inadequate response to monotherapy, and possible first-line therapy in patients with advanced disease (New York Heart Association Functional Class III/IV). In addition, to achieve greater therapeutic response, currently, initial combination therapy at the time of diagnosis is recommended. Moreover, triple combination regimens are also considered in severe PAH, when double therapy fails [27,29].

### 1.4. Non-Pharmacological Therapies

In randomized controlled trials, exercise therapy improves exercise tolerance, functional capacity, and quality of life, with a positive impact on social, emotional, and psychological aspects [3,30]. Therefore, supervised exercise rehabilitation programs are recommended [3]. In addition, it is recommended that patients should avoid excessive physical activity that leads to distressing symptoms such as due to poor gas exchange or improper ventilation. Moreover, exercise programs are not well-stablished and present several limitations based on the gaps in the knowledge of the optimal method, intensity, and duration of the training [31].

Dietary modification is one of the first steps in the treatment of cardiovascular diseases. The routine treatment of systemic arterial hypertension involves dietary interventions for all patients including salt and alcohol restriction; increased consumption of vegetables, fresh fruits, whole grains, soluble fiber, fish, nuts, and olive oil; low consumption of red meat; and consumption of low-fat dairy products [32]. However, the European Society of Cardiology (ESC) and the European Respiratory Society (ERS) Guidelines [3] have not established specific recommendations for dietary habits or nutrient supplementation for PAH.

Interestingly, associations between nutritional factors and PAH have recently been reported in both human epidemiological studies and animal models. Recently, it has been reported that multiple-target nutritional intervention with extra protein, leucine, fish oil, and oligosaccharides can be a new strategy to prevent the pathophysiological alterations such as cardiac and skeletal muscle hypertrophy in PAH [33].

Herein, we focus on the scientific evidence on how the deficit in iron and vitamins C and D as well as other dietary components such as flavonoids may affect the progression of PAH. Finally, the role of the gut microbiota and its interplay with the diet and the host immune system is emerging in multiple cardiovascular and respiratory diseases including PAH. Other dietary factors such as n-3 polyunsaturated fatty acids (PUFAs), vitamin E, melatonin, and coenzyme Q10 may theoretically have an effect in PAH but there is no experimental or clinical evidence to support it and they are not discussed herein.

## 2. Dietary Components with an Impact on PAH

### 2.1. Vitamin C

Vitamin C, also known as ascorbic acid, is a water-soluble vitamin found in several fruits and vegetables. It is required for the activity of several enzymes, involved in tissue repair, important for the immune system function, and functions as an antioxidant. Severe deficit of vitamin C leads to scurvy, causing general weakness, anemia, skin hemorrhages, gum disease, and teeth loss [34,35].

Many studies have shown that oxidative stress is involved in cardiovascular disease [36]. Nitric oxide inactivation by reactive oxygen species is a key event in endothelial dysfunction associated to hypertension and atherosclerosis and other vascular pathologies [37]. On the other hand, oxidation of LDL in the endothelial wall makes these particles more atherogenic and allows them to accumulate in the artery walls [36]. This has led to the wide use of antioxidants including vitamin C to slow the progression of atherosclerosis. However, the meta-analysis of pooled data from randomized controlled trials have concluded that antioxidant vitamin supplementation has no effect on the incidence of major cardiovascular events, myocardial infarction, stroke, total death, and cardiac death [38].

Several case reports have shown that pulmonary hypertension is a complication of scurvy [39,40,41,42]. Elevated mPAP was reversible after the administration of ascorbate. Two possible mechanisms for the involvement of vitamin C deficiency in PAH have been proposed [41]. First, vitamin C increases the availability of endothelial NO that has vasodilatory and antiproliferative capacity [43]. Second, a deficiency of vitamin C can inactivate prolyl hydroxylases, the cellular oxygen sensors, uncoupling hypoxia-inducible factor (HIF) from oxygen control [44]. Uncontrolled HIF activity may lead to activation of pulmonary hypertensive mechanisms [45].

Whether moderate vitamin C deficiency rather than clinical scurvy, which is rare in Western societies, plays a role in PAH is unknown. Moreover, the effect of vitamin C supplements on PAH patients has not been well-addressed yet and there is only preliminary experimental evidence of its effectiveness. For example, a study in broiler chickens has shown that vitamin C reduced the incidence of PAH and the associated muscularization of pulmonary arterioles [46].

### 2.2. Vitamin D

Vitamin D is a fat-soluble vitamin that acts as a steroid hormone. It was discovered as an essential nutrient for the prevention of rickets. Although vitamin D may be obtained from diet, the main source is derived from endogenous synthesis in the skin under the influence of solar ultraviolet B radiation [47]. The inactive precursor synthetized in the skin or diet undergoes a two-step activation process to become biologically active. The first step is the 25-hydroxylation in the liver by CYP2R1 resulting in 25-hydroxyvitamin D_3_ (25(OH)D_3_), also named calcidiol, which has partial activity. The second hydroxylated metabolite is the active 1α, 25-dihydroxyvitamin D_3_ (1,25(OH)_2_D_3_), also called calcitriol, by the 1α-hydroxylase enzyme or CYP27B1 mainly in the kidney [47,48]. Although calcitriol is the active metabolite of vitamin D, calcidiol is the best circulating biomarker of vitamin D status because the calcitriol half-life is shorter than that of calcidiol [47,48]. Calcitriol exerts its functions through the vitamin D receptor (VDR). Similar to other steroid receptor family members, VDR acts as a transcription factor [49]. VDR binds calcitriol with high affinity and specificity and then heterodimerizes with the retinoid-X receptor (RXR). After that, the VDR–RXR complex interacts with the vitamin D response elements on the promoter DNA region of target genes, resulting in changes in gene expression [48,50]. VDR regulates the expression of mRNAs as well as several miRNAs, indirectly regulating the expression of other genes [51].

There is no clear consensus on the definition of vitamin D deficiency; the optimum levels and the dietary requirements are uncertain [52,53]. However, even using conservative thresholds, nowadays, there is a pandemic of vitamin D deficiency [54]. The principal causes of low 25(OH)D_3_ levels are inadequate sun exposure and/or reduced dietary intake [54]. 

Classically, vitamin D deficiency was related to bone diseases. Currently, because of VDR is found is many tissues, such as immune and cardiovascular cells, vitamin D deficiency has also been related to infection, cancer, and respiratory and cardiovascular diseases [53,55,56]. In fact, vitamin D deficiency has been associated with increased all-cause and cardiovascular mortality [57,58]. The discovery of VDR in many tissues that do not participate in calcium and phosphorous homeostasis led to identify a great variety of functions mediated by VDR, such as cell proliferation and differentiation, immunomodulation, and intracellular metabolism, among others [48].

In the context of PAH, there is some basic and clinical evidence suggesting a role for vitamin D in the pathophysiology of the disease. VDR was identified in vascular cells, including endothelial and smooth muscle cells. It is involved in numerous processes of potential relevance in cardiovascular diseases, such as cell proliferation, differentiation, and apoptosis; cell adhesion; oxidative stress; angiogenesis; and immunomodulatory and anti-inflammatory activity [53]. Therefore, it is assumed that vitamin D levels may affect the development of PAH.

To clarify whether vitamin D levels could be involved in PAH progression, Tanaka et al. treated PAH rats with a diet containing 10;000 UI/kg of cholecalciferol [59]. Notably, in this study, they found that vitamin D supplementation in PAH rats improved survival and attenuated some typical features in PAH such as right ventricle remodeling, assessed by Fulton index (ratio of right ventricle weight to left ventricle plus septum weight), and medial thickness of muscular pulmonary arteries. Despite these benefits of vitamin D, cholecalciferol treatment did not decrease pulmonary artery pressure [59]. Moreover, in an in vitro setup, calcitriol treatment inhibited the hypoxia-induced proliferation and migration in rat pulmonary artery endothelial cells (PAEC) via miR-204/TGFβ/Smad signaling pathway. Specifically, calcitriol suppressed the expression of Tgfbr2, α-SMA, and Smad7 and induced miR-204, p21, and Smad2 expression [60]. In the same study, similar results were found in an in vivo rat model. Remarkably, intraperitoneal calcitriol administration (20 mg/kg) partly reversed the rise in mPAP and Fulton index induced by three weeks of hypoxia [60].

In the clinical arena, Ulrich et al. showed that secondary hyperparathyroidism is highly prevalent in PAH patients [61]. Physiologically, decreased serum 25(OH)D_3_ results in increased parathyroid hormone (PTH) levels in order to maintain adequate serum calcium concentrations. Therefore, low vitamin D status in PAH patients could be the reason for the elevated PTH. Later, epidemiological studies demonstrated that vitamin D deficiency is quite prevalent in PAH patients [59,62,63]. In the prospective study carried out by Demir et al., PAH patients presented much lower vitamin D levels (median of 6.79 ng/mL), considered as severe deficit of vitamin D (<10 ng/mL of serum 25(OH)D_3_), than controls (18.76 ng/mL) [62]. In line with this result, Tanaka et al. found that, in a cohort of PAH patients, 39 out of 41 (95.1%) presented vitamin D insufficient and 25 patients (61%) showed deficient levels [59].

The relationship between vitamin D deficiency and PAH prognosis was evaluated. Serum 25(OH)D_3_ levels were negatively correlated with mPAP assessed by right heart catheterization, and a significant positive correlation with cardiac output was found [59]. The potential benefits of vitamin D replacement on clinical outcomes has been also studied [63]. Twenty-two PAH patients were enrolled in a prospective uncontrolled longitudinal study. All PAH patients received cholecalciferol at a dose of 50,000 IU weekly for three months. In addition to the rise of serum 25(OH)D_3_ levels from 14 ± 9 to 69 ± 31 ng/mL, remarkably, vitamin D supplements improved the 6-min-walk-distance (6MWD) test by around 80 m and right ventricle size. Mean PAP estimated by echocardiography was reduced from 79 ± 25 to 69 ± 23 mmHg but this effect did not reach statistical significance. Pro-BNP (pro-Brain Natriuretic Peptide) and functional class were also unchanged after vitamin D therapy [63].

All these data point to beneficial effects of vitamin D in PAH. However, the therapeutic use of vitamin D in this context has not been validated in randomized clinical trials. Nevertheless, given the high prevalence of vitamin D deficiency associated to PAH, it seems reasonable that serum vitamin D levels should be regularly assessed in these patients. Vitamin D supplements should be used to prevent bone diseases in any subject showing moderate or severe deficiency. Whether the symptoms, quality of life, and prognosis of patients with PAH improve after restoring vitamin D levels is unclear. Vitamin D supplementation has been used in other conditions. For instance, vitamin D supplements succeeded in respiratory diseases, decreasing the incidence of asthma [64] and chronic obstructive pulmonary disease (COPD) [65] exacerbations in patients with baseline 25(OH)D_3_ levels lower than 25 nmol/L [65]. On the contrary, vitamin D supplementation has failed in other pathologies. In several of these latter studies, baseline vitamin D levels have not been taken into account [66,67,68].

In view of these results, it is plausible that vitamin D deficiency in combination with others risk factors could aggravate PAH. Vitamin D deficiency per se does not cause PAH. This is consistent with the fact that vitamin D deficiency is very prevalent in the population [54] while PAH is a rare disease. Therefore, further research is necessary to investigate the harmful effects of vitamin D deficiency in the pathogenesis of PAH and the efficacy and safety of vitamin D treatments.

### 2.3. Iron

Iron is essential in several physiological processes, including oxygen delivery and energy metabolism. In fact, about 70% of iron is bound to hemoglobin and around 5–10% is found in myoglobin. Serum ferritin is the most specific indicator used in laboratories for evaluating iron stores. Ferritin levels below 30 ng/mL are considered iron deficiency with or without anemia [69,70]. Circulating soluble transferrin receptor levels is another biomarker of iron deficiency. Iron deficiency is the most common cause of anemia worldwide and it is particularly common in specific chronic diseases such as heart failure or chronic renal diseases [71].

Recent data indicate that iron deficiency is also prevalent in patients with idiopathic PAH (IPAH) and it correlates with disease severity [72,73,74]. In fact, anemia is also an indicative of poor prognosis [75,76]. For the first time, Ruiter et al. [73] reported that around of 40% IPAH patients present iron deficiency and it is associated with decreased exercise capacity, assessed by the 6MWD test without anemia. Similar results were found by Yu in patients with PAH associated with congenital heart disease [77]. The significantly decreased 6MWD suggests that iron is essential in maintaining exercise performance. The authors speculated that iron deficiency might impair oxygen transport and delivery and finally disturb muscle oxygen homeostasis. Consequently, the clinical manifestation is shorter 6MWD. Interestingly, restoring iron levels in patients with chronic left heart failure significantly improves 6MWD and New York Heart Association (NYHA)-Functional Class [78,79].

Although epidemiological data show iron deficiency in PAH, the physiological contribution of iron in PAH is unknown. Few studies have been carried out in this context [80,81,82,83]. Variation in iron availability without anemia can affect pulmonary vascular tone. Intravenous infusion of iron attenuated the increased in mPAP in response to sustained hypoxia in 16 healthy volunteers with normal iron levels [83]. Likewise, acute iron depletion exacerbates PAP and pulmonary vasoconstrictive response to hypoxia condition [83]. In line with these results, in individuals exposed to high altitude, PAH may be attenuated by iron supplementation [84].

After four weeks of iron deficient diet, rats present vascular remodeling in resistance pulmonary arteries and PAH. These vascular changes were accompanied by activation of HIF, STAT3, and mitochondrial dysfunction. In addition, in this study, mPAP and pulmonary vascular muscularization was reversed by intravenous iron therapy [80]. Transferrin-1 receptor (TfR1) knock-out mice show protection against the development of hypoxia-induced PAH. Similarly, downregulation of TfR1 in vitro also inhibits human PASMC proliferation [85]. Moreover, recently, Lakhal-Littleton et al. demonstrated that intracellular iron deficient in PASMC induces PAH in mice via increasing expression of ET-1 [86].

The cause of the increased prevalence of iron deficiency in PAH is not completely clear. Iron deficiency can be related to reduced intake, impaired uptake, or increased loss of iron. Some authors postulated that the predominance of PAH in women vs. men could be due to a higher prevalence of iron deficiency in premenopausal women compared to postmenopausal women and men [77,87,88]. However, ferritin levels and circulating soluble transferrin receptor levels did not differ with gender or age in a large cohort of IPAH patients [72,73]. On the other hand, it is interesting that only a small proportion of IPAH responded to oral iron therapy, suggesting that, at least in these group of patients, a disturbance in iron absorption could be responsible for iron deficiency [73,89]. In line with this theory, elevated hepcidin levels were found in IPAH patients [72]. Hepcidin is a hormonal inhibitor of the intestinal absorption of dietary iron synthesized by the liver, which is elevated in inflammation [90]. However, plasma hepcidin concentration did not correlate with IL-6 levels, suggesting that, at least in these cohorts of IPAH patients, raised hepcidin levels were not due to inflammation. Of particular interest is BMP signaling. In vitro, *BMPR2* downregulation by a short interfering RNA increased hepcidin production. Rhodes et al. speculated that BMPR2-heritable PAH might be associated with more severe iron deficiency due to increased hepcidin levels [72].

All this evidence suggests that intravenous iron replacement could be a potential treatment in PAH patients [91,92], improving hemodynamic and clinical outcomes.

### 2.4. Flavonoids and Other Polyphenols

Polyphenols are a large group of plants metabolites commonly present in the human diet, specifically in vegetables, fruits, and beverages. Flavonoids comprise the major group of polyphenolic compounds. They are chemically characterized, sensu stricto, by the presence of a skeleton of 2-phenyl-4H-1-benzopyrane [93]. Isoflavonoids, neoflavonoids, chalcones, and aurones are related compounds often considered flavonoids as well. Other important polyphenols include stilbenoids.

Many studies have analyzed the influence of polyphenols in human heath [94], and more especially its positive role against cardiovascular diseases [95,96]. In addition to their antioxidant action, they also present vasodilator, antithrombotic, antiapoptotic, anti-inflammatory, hypolipidemic, and antiatherogenic effects, associated with decreased cardiovascular risk [96]. The flavonoids present in fresh fruits, vegetables, and wine are considered major contributors to the antihypertensive effects of these foodstuffs. In particular, the effects of the flavonoid quercetin, the isoflavonoid genistein, and the stilbenoid resveratrol have been studied in animal models of PAH and are reviewed herein.

Resveratrol is found in red wine, grapes, and berries. This polyphenol has been shown to attenuate right ventricular systolic pressure and pulmonary artery remodeling in monocrotaline-induced PAH in rats [97]. Moreover, this study demonstrated that resveratrol treatment (25 mg/kg per day) improved pulmonary endothelial function, assessed by increased eNOS expression, and decreased oxidative stress due to decreased of NADPH oxidase activity. Resveratrol also reduced the inflammatory cytokines IL-1β, IL-6, and TNFα, and inhibited PASMC proliferation [97]. Therefore, resveratrol exerted anti-oxidant, anti-inflammatory, and anti-proliferative effects, reducing the main hallmarks of PAH. It was speculated that ROS scavenging mediated by resveratrol may be the central process of these pleiotropic actions [98]. Several experiments have been performed to elucidate the underlying mechanisms. Chen et al. found that in vitro resveratrol treatment attenuated the hypoxia-induced proliferation in human PASMC by the inhibition of arginase II. The inhibitory effect of resveratrol on arginase II was PI3K-Akt signaling pathway-dependent [99]. Similar results were found in rat PASMC [100], in hypoxic pulmonary hypertension rats [101], and in monocrotaline-induced PAH [102].

Quercetin is probably the most widely distributed in foods and best studied flavonoid. Multiple studies have highlighted its biological activity to reduce arterial blood pressure in both human and experimental systemic hypertension [103,104]. Several animal models have also been used to examine the protective effect of quercetin in PAH. The first report analyzed the effects of quercetin as a preventive strategy for PAH (100 mg/kg from the day after monocrotaline infusion) [105]. Consecutively, our group investigated the therapeutic role of quercetin in PAH induced by monocrotaline in rats (10 mg/kg once daily from Day 21 after PAH was established) [106]. In both studies, the authors found that quercetin administration significantly alleviated mPAP, right ventricular hypertrophy, and pulmonary artery remodeling. Furthermore, quercetin treatment significantly increased survival in monocrotaline rats. However, classic biomarkers of PAH, such as endothelial dysfunction, pulmonary artery hyperresponsiveness to 5-HT, and downregulation of BMPR2 and Kv1.5, were unaffected by quercetin [106]. These were unexpected results because quercetin has been widely reported to improve endothelial function in systemic arteries in in vivo and in vitro experiments [104,107]. Our group also demonstrated that quercetin exerted vasodilator effect in isolated pulmonary arteries, induced apoptosis and inhibited cell proliferation in PASMC [106]. The mechanism involved in the antiproliferative effects in both PASMC and endothelial cells seem to involve AKT [106,108,109], FOXO1-mTOR [110], and altered Bax/Bcl-2 ratio [108,111].

Genistein is an isoflavone abundant in soybeans. It has been widely used as a phytoestrogen substitute for hormone replacement therapy in postmenopausal women [112]. Genistein consumption is thought to reduce the incidence or severity of cardiovascular disease and of some forms of cancers [113]. It is a wide spectrum tyrosine kinase inhibitor (TKI) and it is well-known that tyrosine kinase inhibitors play an important role in the control of pulmonary vascular tone. Genistein can behave as an antioxidant and improves endothelial function in systemic and pulmonary arteries from several models of cardiovascular disease, through increasing endothelial NO synthase levels, restoring NO-mediated PA relaxation, reducing vascular superoxide production or decreasing angiotensin II receptor [114,115,116]. The vasodilator effect of genistein has also been studied in isolated pulmonary arteries precontracted by 5-HT [21] and ET-1 [117]. The activation of 5-HT_2A_ receptors inhibits K_V_ currents, and genistein treatment prevented this effect in rat PASMC [21]. In PA from chronic hypoxia rats, the contraction induced by ET-1 appeared to be mediated by the activation of tyrosine kinase, and genistein reduced the ET-1-induced response [117]. All these effects make genistein a potential therapy for PAH. In the rat model of PAH induced by monocrotaline, genistein both prevents [118] and reverses [116] the increased PAP. Moreover, genistein significantly improved pulmonary vascular remodeling, right ventricular function, and survival. It also inhibited human PASMC proliferation in vitro [116]. In addition, genistein also ameliorated pulmonary hemodynamics and vascular remodeling in a rat model of hypobaric hypoxia [119]. Some authors suggested that the mechanism underlying genistein-improved main characteristics of PAH is mediated through the improvement of PI3K/Akt/eNOS signaling pathway [119,120]. In addition, genistein also potently attenuates hypoxia-induced hypertrophy of PASMC through estrogen receptor and β-adrenoreceptor signaling [121].

### 2.5. Microbiota

The human gut is a bacterial ecosystem that harbors >100 trillion microbial cells and presents a symbiotic relationship with the host. Gut microbes provide help with digestion, promote gut immunity, and prevent the colonization of pathogens, while the host supplies them with a favorable environment for survival. A healthy gut microbiome is characterized in terms of diversity and richness as well as its stability and resistance to any perturbation. In contrast, gut dysbiosis is any disruption of the normal balance between the gut microbial community and the host, which can result in several diseases [122]. Gut dysbiosis is typically characterized by a lower diversity and richness of the microbial communities, an increase in Firmicutes to Bacteroidetes ratio (F/B), and altered short chain fatty acids (SCFA) producing bacteria, with an increase in lactate-producing bacteria and a decrease in acetate- and butyrate-producing bacteria [123,124]. In recent years, a growing body of evidence points to a relationship between gut dysbiosis and many diseases, including essential hypertension [124,125], obesity [126,127], inflammation [128] neurologic disorders [129], and pulmonary hypertension [130].

The diet is a critical regulator of the composition and function of the microbiota [131]. Multiple studies have focused on the effects of macronutrients (fat, carbohydrate, and protein) on the gut microbiome. Other dietary components such as soluble or insoluble fibers may be important as well [132,133]. Moreover, several food components are substrates for bacterial enzymes. These enzymatic processes lead to the production of other byproducts which can be absorbed in the gut. Importantly, SCFAs, particularly butyric and acetic acid, which derive mainly from the bacterial fermentation of fiber, are considered to promote cardiovascular health. In contrast, trimethylamine-N-oxide (TMAO), a metabolite produced by the gut microbiota from choline, betaine, and carnitine, which are abundant in meat, eggs, and fish, is associated with excess risk of heart disease [134].

In addition, it has been reported that some dietary components such as sweeteners, minerals, and vitamins can modify the microbiota. Remarkably, some of the nutrients with an impact on PAH progression, as described above, such as iron and vitamin D deficiency as well as quercetin and resveratrol significantly affect the intestinal microbiota [135,136,137]. Therefore, besides the aforementioned mechanisms of action of these dietary components, the changes in the gut microbiota may also be responsible of the actions of iron, vitamin D, or polyphenols. On the contrary, the composition of microbiota may affect the absorption of calcium, phosphate, iron, and zinc. Moreover, in addition to dietary sources of water-soluble vitamins, the microbiota can also synthetize some of these vitamins [132].

The role of the diet on the microbiome in the context of PAH is not known. However, it could be speculated that part of the effects of the dietary factors mentioned above in PAH might be due to changes in the microbiota. We demonstrated for the first time that there are several changes in the gut microbiota in PAH [130]. In a rat model of PAH induced by a single dose of Sugen5416 plus chronic hypoxia for two weeks, we found two main hallmarks of gut dysbiosis: a three-fold increase in F/B ratio, driven by a decrease in all Bacteroidetes families in PAH animals (2–10-fold decrease) and no changes in Firmicutes abundance. Furthermore, feces from PAH rats present a decreased in acetate-producing bacteria, accompanied by a reduced serum acetate, without changes in butyrate and lactate producing bacteria [130]. In contrast, we did not find global differences in microbial diversity and richness, as happened in other diseases [124]. Although this study is preliminary, it indicates that the abnormalities in the gut microbiota observed might play a pathophysiological role in the development and/or progression of PAH, rather than being a consequence. Likewise, Wedgwood et al. also suggested that intestinal dysbiosis may impact on distal organs including the lung, contributing to the development of PH [138]. In this study, rat pups with PH induced by postnatal growth restriction (PNGR) present gut dysbiosis and the probiotic treatment attenuates PNGR-induced PH. Considering these results, the authors suggested that PH is in part driven by the alteration of the gut microbiome [138].

It is tempting to speculate that changes in intestinal microbiota and circulating microbial products can contribute to PAH. Thenappan et al. suggested that gut dysbiosis might be involved in perivascular inflammation in the early development of PAH [139]. Gut dysbiosis can result in increased gut permeability, allowing bacteria and/or bacterial products translocation, with an increase in plasma bacterial lipopolysaccharide (LPS), the main ligand for toll-like receptor 4 (TLR4). TLR4 activation has been implicated in the pathogenesis of PAH [140]. Ranchoux et al. demonstrated that bacterial translocation occurs in PAH, suggesting a gut-lung cross-talk, in which TLR4 antagonists are plausible to be effective at disrupting this circle [141]. Gut dysbiosis also produces a pro-inflammatory environment, increasing IL-17 secretion and a downregulation of Treg cells [142]. Likewise, an increase in Th17 cells and a deficiency in normal Treg cells are observed in PAH patients, promoting vascular remodeling [26,143]. In addition to platelets, serotonin (5-HT) is also stored and produced in enterochromaffin cells. Thus, gut microbiota plays a key role in regulating 5-HT levels at colon and serum. Notably, clinical and experimental PAH showed elevated serum 5-HT levels. It is well-known that 5-HT promotes pulmonary artery remodeling, PASMC proliferation, and constriction of pulmonary arteries through the 5-HT_1B_ receptor [21,144].

## 3. Conclusions

**Figure 1:**
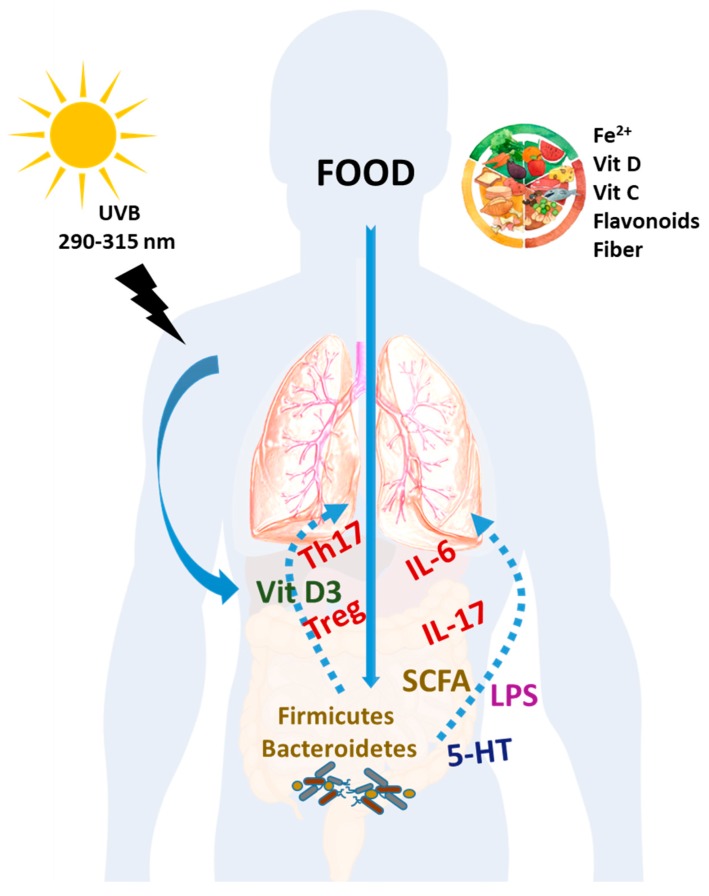
Impact of nutrition in PAH. Dietary components such as Fe^2+^, vitamins C and D, flavonoids and other related polyphenols, and fiber as well as vitamin D obtained from the exposure to sunlight may have a positive impact the quality of life and prognosis of PAH patients. Each dietary factor may have its own mechanism of action. However, part of the effects of these nutrients may be related to their effect on the immune system with restoration of T cells and cytokines, changes in the microbiota and their bacterial products, and bacterial translocation.

Although there have been important advances in the knowledge of the pathophysiology of PAH, it remains a debilitating, limiting, and rapidly progressive disease. Targeted nutritional and lifestyle interventions could have a great clinical importance (Figure 1). Vitamin D and iron deficiency are worldwide health problems of pandemic proportions. Notably, these nutritional alterations are largely more prevalent in PAH patients than in the general population and there are several pieces of evidence suggesting that they may trigger or aggravate the disease progression. However, to date, most of this evidence is based on observational studies, animal models, and small series of uncontrolled trials. Therefore, robust randomized clinical trials are required to establish cause–effect relationships. In the meantime, it seems reasonable to study the nutritional status of all PAH patients with particular emphasis on vitamins C and D and iron. Severe nutritional deficiencies leading to scurvy, osteoporosis, or ferropenic anemia must be corrected using the appropriate supplements. Based on the above discussed evidence, the correction of these nutritional defects may be expected to have additional positive impact on the severity of the disease, the quality of life, and the prognosis of the patients.

The possible positive effects of the polyphenols quercetin, resveratrol, and genistein in PAH remain to be determined in clinical trials. The use of supplements containing these polyphenols cannot be recommended at this stage. However, given the encouraging effects of fruits and vegetables on cardiovascular health with particular impact on systemic hypertension, it seems reasonable to stimulate PAH patients to adhere to diets rich in these foods.

The role of gut dysbiosis in the pathogenesis of PAH has not been firmly established. At present, no recommendations directed to modify the gut or the lung microbiota can be established. However, if the role of dysbiosis is confirmed, several interventions may be implemented to correct or compensate the altered microbial ecosystem including the use of specific bacterial strains (probiotics), fiber and dietary polyphenols (i.e., prebiotics), fecal transplantation, antibiotics, and beta-adrenergic antagonists or replacing the deficit in specific SCFAs (e.g., acetate).
